# Use of contraceptives, high risk births and under-five mortality in Sub Saharan Africa: evidence from Kenyan (2014) and Zimbabwean (2011) demographic health surveys

**DOI:** 10.1186/s12905-018-0666-1

**Published:** 2018-10-24

**Authors:** Admire Chikandiwa, Emma Burgess, Kennedy Otwombe, Lucy Chimoyi

**Affiliations:** 1 Wits RHI, Faculty of Health Sciences, University of the Witwatersrand, 22 Esselen St, Hillbrow, Johannesburg, 2001 South Africa; 2 Perinatal HIV Research Unit, Faculty of Health Sciences, University of the Witwatersrand, Johannesburg, South Africa; 3 The Aurum Institute, 29 Queens Road, Parktown, Johannesburg, 2194 South Africa; 4 School of Public Health, Faculty of Health Sciences, University of Witwatersrand, Johannesburg, South Africa

**Keywords:** Contraceptive use, High risk births, Kenya, Maternal mortality, Under-five mortality, Zimbabwe

## Abstract

**Background:**

Increasing uptake of modern contraception is done to alleviate maternal and infant mortality in poor countries. We describe prevalence of contraceptive use, high risk births, under-five mortality and their risk factors in Kenya and Zimbabwe.

**Methods:**

This was a cross-sectional analysis on DHS data from Kenya (2014) and Zimbabwe (2011) for women aged 15–49. Geospatial mapping was used to compare the proportions of the following outcomes: current use of contraceptives, high-risk births, and under-5 mortality at regional levels after applying sample weights to account for disproportionate sampling and non-responses. Multivariate risk factors for the outcomes were evaluated by multilevel logistic regression and reported as adjusted odds ratios (aOR).

**Results:**

A total of 40,250 (31,079 Kenya vs. 9171 Zimbabwe) women were included in this analysis. Majority were aged 18–30 years (47%), married/cohabiting (61%) and unemployed (60%). Less than half were using contraceptives (36% Kenya vs. 41% Zimbabwe). Spatial maps, especially in the Kenyan North-eastern region, showed an inverse correlation in the current use of contraceptives with high risk births and under-5 mortality. At individual level, women that had experienced high risk births were likely to have attained secondary education in both Kenya (aOR = 5.20, 95% CI: 3.86–7.01) and Zimbabwe (aOR = 1.63, 95% CI: 1.08–2.25). In Kenya, high household wealth was associated with higher contraceptive use among both women who had high risk births (aOR: 1.72, 95% CI: 1.41–2.11) and under-5 mortality (aOR: 1.66, 95% CI: 1.27–2.16). Contraceptive use was protective against high risk births in Zimbabwe only (aOR: 0.79, 95% CI: 0.68–0.92) and under-five mortality in both Kenya (aOR: 0.79, 95% CI: 0.70–0.89) and Zimbabwe (aOR: 0.71, 95% CI: 0.61–0.83). Overall, community levels factors were not strong predictors of the three main outcomes.

**Conclusions:**

There is a high unmet need of contraception services. Geospatial mapping might be useful to policy makers in identifying areas of greatest need. Increasing educational opportunities and economic empowerment for women could yield better health outcomes.

**Electronic supplementary material:**

The online version of this article (10.1186/s12905-018-0666-1) contains supplementary material, which is available to authorized users.

## Background

The third Sustainable Development Goal of the United Nations aims for healthy living and well-being for all and targets reduction of maternal mortality ratio (MMR) to under 70 deaths per 100,000 live births and under-5 mortality rate (U-5MR) to below 25 per 1000 live births respectively [1]. More effort will be required in developing regions and especially Sub-Saharan Africa [SSA] where MMR remains way higher than in developed regions [2] and eight of ten deaths in children is under the age of five [3].

Increasing access to contraceptive methods has been recommended for the reduction of MMR and U-5MR [4]. The impact of contraceptive use on mortality is mediated through factors that include early births (mother’s age is below 18 years), giving birth late (mother’s age is above 34 years), short period of time between births (less than 2 years), and high parity rates (i.e. > 3 children) which are often referred to as high risk births as they represent increased chance of mortality to both the mother and child [5–7].

In SSA, the contraceptive prevalence rates have remained low, and the region continues to face high MMR and U-5MR [8]. The region is characterized by high fertility and often unwanted pregnancies, which could be due to low contraceptive usage [9]. Available data from Kenya and Zimbabwe has suggested that child mortality rates are related to maternal factors which include age at childbirth, spacing of births and parity [10, 11].

Prior research from SSA shows that contraceptive coverage is further influenced by a number factors including: medical barriers such as accessibility of the services, health care workers attitudes, medical and regulatory guidelines; [12] cultural norms such as the role of women in decision making, place of residence (i.e. rural vs urban), religious and cultural beliefs [13] as well as individual level factors which include age, education, employment, marital status, parity [14]. Community level factors which include, illiteracy rate and poverty, have been shown to influence uptake of healthcare services especially in rural areas [15]. There are also other factors that influence MMR and U-5MR beyond contraception like accessing education and quality of antenatal care [16, 17]. However, there is scarcity of data exploring usage of contraceptives and outcomes such as high risk births as well as under-five mortality from geospatial distributions and contextual community factors using multi-country data. To address this gap, we conducted a comprehensive comparative cross-sectional analysis using the most recent Demographic Health Survey (DHS) data from Kenya (2014) and Zimbabwe (2011) at the time, to investigate the (i) spatial distribution of contraceptive coverage, prevalence of high-risk births and under-five mortality, and (ii) correlates of contraceptive usage at individual and community level among women experiencing under-five mortality or high risk births.

## Methods

### Study design, population and sample size

This population-based study used data from Kenya DHS 2014 and Zimbabwe DHS 2011 focussing on females in the child-bearing age of 15–49 years. The DHS has a two-stage cluster sampling technique using two strata; place of residence (rural/urban) or administrative regions. A sample of 1612 (Kenya) and 406 (Zimbabwe) clusters was drawn from previous census by the Kenya National Bureau of Statistics and the Zimbabwe National Statistics Agency respectively. Each cluster randomly selected from an enumerated area (EA) contained a total of 25 (Kenya) and 27 (Zimbabwe) EAs. We used individual weighted data on 31,079 and 9171 women and their associated child records from Kenya and Zimbabwe, respectively. During analysis, we used variables with up to 85% of their observations available. We also created composite variables that combined information from other variables as described in the next section.

### Data collection and variables

Information on sampling techniques and procedures applied for data collection in both countries have been published in the final DHS reports [18, 19].

#### Outcome variables

Three outcomes were investigated in the analysis. Primarily, modern contraception use, defined as reporting the use of any modern contraception by women in a union aged 15–49. As per DHS definition, modern contraception included the following: female or male sterilisation, intrauterine contraceptive devices, hormonal methods such as oral contraceptive pill, injectables and implants, barrier methods like female or male condoms, diaphragms, as well as spemicides and lactational amenorrhoea. Among women who had ever reported a live birth in their lifetime we also assessed: under-five child mortality, defined as death of a child aged five years and below, and high-risk births, defined as giving birth below 18 years or above 35 years of age, of high birth order (> 3 children), or with short spacing (< 2 year-gap between children). All these three outcomes were binary (i.e. yes which was coded as 1 or no coded as 0).

#### Explanatory variables

Individual and community characteristics were examined for possible associations with contraceptive use (in women that experienced high risk births under-five child mortality), high-risk births and under-five child mortality. Characteristics were chosen because previous studies have found them to be important factors [13, 15]. Individual variables included age of the woman, level of education (primary/secondary/beyond secondary), employment status (yes/no), marital status (never/previously/currently married), number of children and birth order, wealth index (high/middle/low) and religion. Community-level variables were calculated using the primary sampling unit (PSU) of the data. Community characteristics were selected to consider the socio-economic status of the community. This was done by combining four factors; place of residence (rural/urban), proportion of illiteracy, poverty and unemployment. Place of residence was a direct level independent variable whereas proportion of illiteracy, poverty and employment were aggregates of individual independent variables. The aggregates were proportions generated within PSUs by dividing the prevalence of each factor (i.e. those with any employment) in the PSU by the number of women in the PSU [20].

### Data analyses

We analysed data using Stata 14.1 software [21] and choropleth map creation using ArcMap 10.4 [22]. The statistical methods used are adopted from similar work conducted in Burkina Faso by Maiga et al. [8].

#### Descriptive analysis

Descriptive statistics such as medians and interquartile ranges (IQR) for continuous measures were determined for participant and community characteristics. Sample weights were applied on the estimation of proportions and frequencies to adjust for disproportionality due to non-response. Cross tabulations were conducted on categorical variables to determine their association. Choropleth maps were used to display the geographical distribution of the outcome variables.

#### Measures of association (fixed effects)

Risk factors for high-risk births, usage of contraceptives among women experiencing high risk births and under-five mortality were assessed by multilevel logistic regression models. These were fitted under univariate and multivariate models at individual and community levels. Odds ratios and their 95% confidence bands were used to assess the measure of association. Interpretations in the manuscript were based on the adjusted Odds ratios (aOR) from the multivariate models.

#### Assessing variation between communities using the random effects model

Variability within communities was evaluated using the random effects model. Additionally, variability changes in the empty and consecutive model were assessed proportionately using the proportional change in community variance (PCV) [20].

#### Modelling approaches

The logistic regression model was fitted due to the binary nature of the dependent variable whereas the hierarchical nature of the DHS data allowed for the fitting of multilevel models in which individual women (level 1) were nested in their communities (level 2). This allowed for the modelling of relationships between independent variables on the dependent outcome. In order to decompose the total variance shared at the individual and community levels, an empty model was fitted. All the individual-level factors were included in model 2 and community-level factors into model 3 while the model 4 comprised individual-level and community-level factors.

#### Model fitness and precision

In the individual and community level multivariate regressions, the log-likelihood and Akaike Information Criterion (AIC) were used to assess how well the model fitted, taking the one with the smallest value as the best.

## Results

### Descriptive analysis

**Fig. 1:**
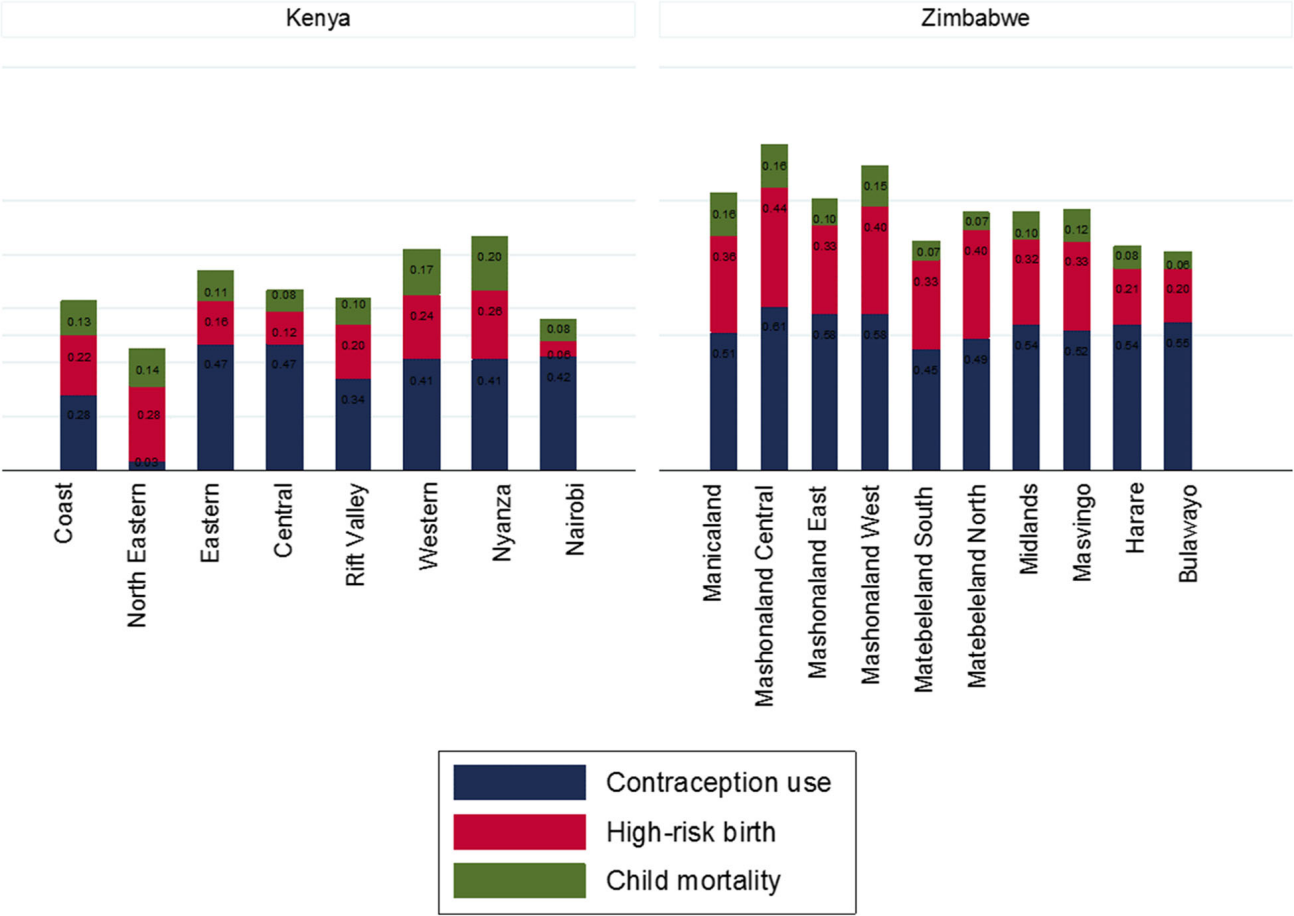
Distribution of regional contraceptive coverage, prevalence of high risk births and under-five mortality in Kenya and Zimbabwe

**Fig. 2:**
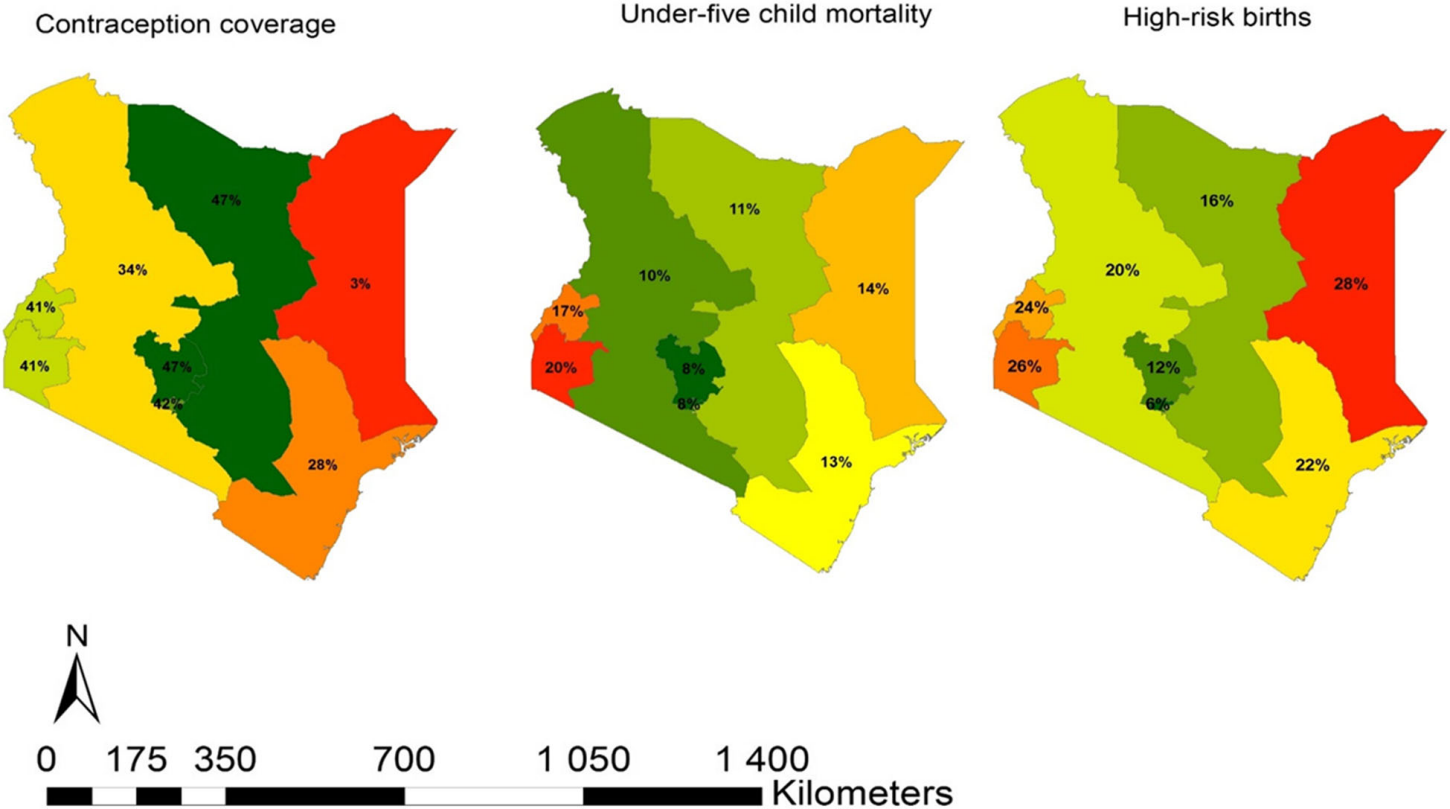
Spatial distribution of regional contraceptive coverage, prevalence of high-risk births and under-five mortality in Kenya

**Fig. 3:**
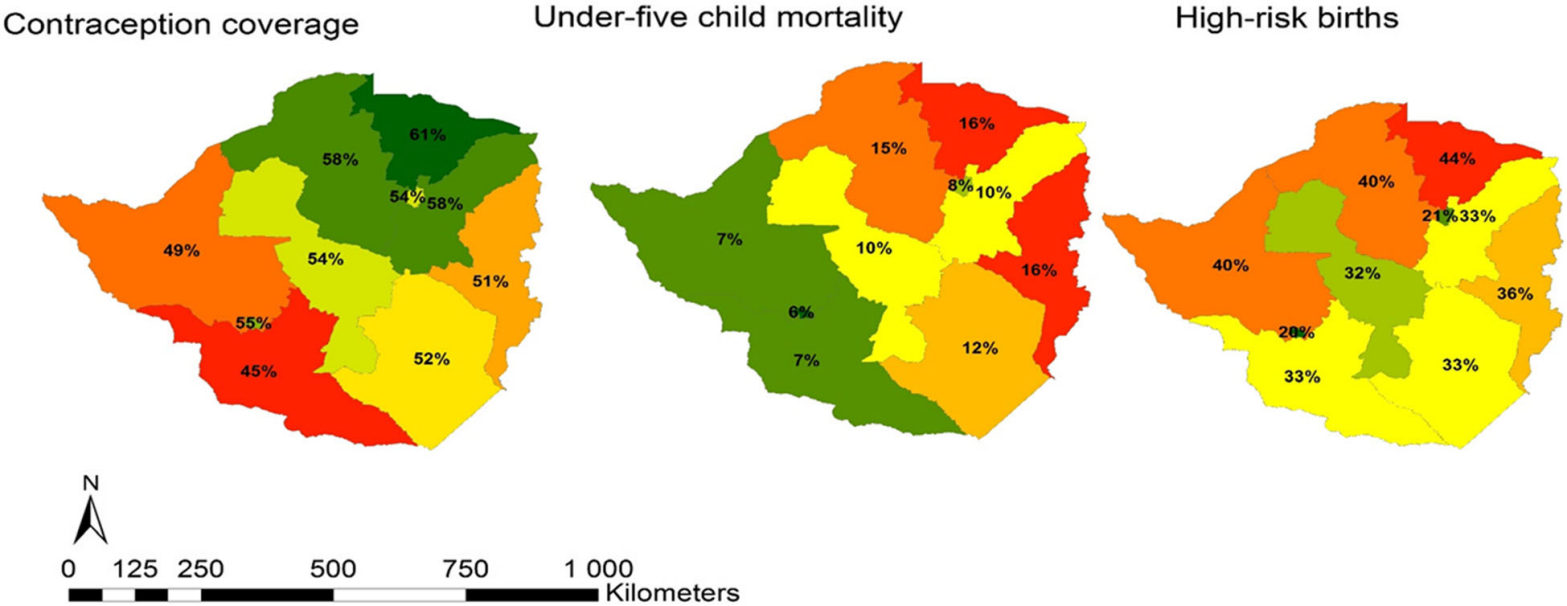
Spatial distribution of regional contraceptive coverage, prevalence of high risk births and under-five mortality in Zimbabwe

A total of 40,250 women were eligible for inclusion in this study; 31,079 from Kenya and 9171 from Zimbabwe. Majority were of age 18–30 years (48% vs. 46%), married/living with a partner (61% vs. 61%) and unemployed in the past 12 months (62% vs. 59%) in Kenya and Zimbabwe (Table 1). Less than half of the women reported current contraceptive use (36% and 41%) where the main route of administration was injections (18%) and oral (26.4%) contraceptives in Kenya and Zimbabwe respectively. Majority of the women in Kenya had attained primary education (50%) while Zimbabweans had at least attained secondary education (69%). Distribution of contraception coverage, high-risk birth and under-five mortality from both countries are presented in Figs. 1, 2 and 3. In Kenya, low contraceptive coverage was reported in North-Eastern province (3%); high under-five child mortality in Nyanza province (20%); and more high-risk births in North-Eastern province (28%). The North-Eastern province which had the lowest coverage for contraceptive (3%), had a higher prevalence of under-five child mortality (14%) and the highest prevalence of high-risk births. In Zimbabwe, there was low contraceptive usage in Matabeleland South (45%); high under-five child mortality in Mashonaland Central (16%) and Manicaland (16%); and more high-risk births in Mashonaland Central (44%). Mashonaland central had the highest contraceptive coverage (61%) and also the highest prevalence of under-five child mortality (16%) and high-risk births (44%). Further comparisons in these countries by rural-urban divide are presented in Additional file 1: Figure S1, Additional file 2: Figure S2, Additional file 3: Figure S3, Additional file 4: Figure S4.

**Table 1:** Baseline demographic characteristics of women who have ever given birth in the Kenyan (*N* = 31,079) and Zimbabwean (*N* = 9171) DHS

Characteristic	Kenya	Zimbabwe
	N (%)	N (%)
Individual level factors		
Age categories		
< 18 years	3769 (12.1)	1603 (17.5)
18–30 years	14,921 (48.0)	4181 (45.6)
> 30 years	12,389 (39.9)	3387 (36.9)
Marital status		
Married/ living with partner	19,036 (61.3)	5578 (60.8)
Divorced/ separated/widowed	3468 (11.1)	680 (7.4)
Never in union	8575 (27.6)	2913 (31.8)
Employment in past 12 months?		
Yes	5628 (38.2)	3776 (41.2)
No	9111 (61.8)	5395 (58.8)
Modern contraceptive use		
Yes	11, 025 (35.5)	3711 (40.5)
No	19,047 (61.3)	5388 (58.8)
Modern Contraceptive type		
None	19,047 (61.3)	5388 (58.8)
Oral	1439 (4.6)	2423 (26.4)
IUD	614 (2.0)	15 (0.2)
Injection	5516 (17.8)	594 (6.4)
Condoms	797 (2.6)	358 (3.9)
Implants	1969 (6.3)	226 (2.5)
Sterilization	655 (2.1)	58 (0.6)
Other	1, 042 (3.4)	82 (0.9)
Mothers education level		
None	4183 (13.5)	224 (2.4)
Primary	15,613 (50.2)	2650 (28.9)
Secondary or higher	11, 283 (36.3)	6297 (68.7)
Household wealth index		
Low	13,232 (42.6)	3292 (35.9)
Middle	5946 (19.1)	1589 (17.3)
High	11,901 (38.3)	4290 (46.8)
Mothers religion		
None	506 (1.6)	589 (6.4)
Roman Catholic	6229 (20.0)	764 (8.3)
Protestant	20,072 (64.6)	6757 (73.7)
Muslim	4, 161 (13.4)	40 (0.4)
Other	11 (0.4)	1021 (11.3)
Number of children		
0	7834 (25.2)	2446 (26.7)
1	4426 (14.2)	1722 (18.8)
2–3	8856 (18.5)	3042 (33.2)
> 3	9963 (32.1)	1961 (21.4)
High risk births		
No	16,742 (72.0)	3729 (55.5)
Yes	6503 (28.0)	2996 (44.5)
Under 5 child mortality in the last 5 years		
No	19,307 (83.1)	5745 (85.4)
Yes	3938 (16.9)	980 (14.6)
Community level factors		
Place of residence		
Urban	11, 614 (37.4)	3437 (37.5)
Rural	19,465 (62.6)	5734 (62.5)
Proportion of unemployment*		
0%	29,958 (96.4)	151 (1.7)
Up to 10%	812 (2.6)	836 (9.1)
Up to 50%	309 (1.0)	7,489,981.7)
Above 50%	–	695 (7.6)
Proportion of illiteracy		
0%	3193 (10.3)	7972 (57.0)
Up to 10%	6251 (20.1)	1185 (12.9)
Up to 50%	16,595 (53.4)	4 (0.1)
Above 50%	5040 (16.2)	–
Poverty level		
0%	5778 (18.6)	4290 (46.8)
Up to 10%	2282 (7.3)	4, 875 (53.2)
Up to 50%	10,652 (34.3)	6 (0.1)
Above 50%	12,367 (39.8)	–

*52.6% missing information

### Multivariate analysis

#### Individual level factors

Table 2 presents risk factors for contraceptive usage in women stratified by high-risk birth and under-five mortality in both countries. At individual level in Kenya among women who have experienced high-risk births, there were higher odds for contraceptive use in those who were married or living with a partner (aOR: 2.98, 95% CI: 1.67–5.29), had primary (aOR: 4.57, 95% CI: 0.36–5.77) and secondary or higher level of education (aOR: 5.20, 95% CI: 3.86–7.01). Those > 34 years of age had lower odds (aOR: 0.57, 95% CI: 0.48–0.68). In Zimbabwe, primary (aOR: 1.53, 95% CI: 1.04–2.25) and secondary or higher level of education (aOR: 1.63, 95% CI: 1.08–2.47) and employment in the past 12 months (aOR: 1.27, 95% CI: 1.07–1.51) was associated with higher odds for modern contraceptive use. Those married or living with partner (aOR: 0.29, 95% CI: 0.21–0.40) and previously married (aOR: 0.19, 95% CI: 0.14–0.26) experienced lower odds.

**Table 2:** Individual, household and community level factors associated with contraceptive use in women who have experienced high-risk births and under-five child mortality

	Kenya				Zimbabwe			
	High-risk birth (*n* = 6503)		Under-five mortality (*n* = 3938)		High-risk birth (*n* = 2996)		Under-five mortality (*n* = 980)	
Characteristic	Univariate OR (95% CI) *p*-value	Multivariate aOR (95% CI) p-value	Univariate OR (95% CI) p-value	Multivariate aOR (95% CI) p-value	Univariate OR (95% CI) p-value	Multivariate aOR (95% CI) *p*-value	Univariate OR (95% CI) *p*-value	Multivariate aOR (95% CI) *p*-value
Individual level factors								
Age categories								
18–34 years	1.00	1.00	1.00	1.00	1.00	1.00	1.00	1.00
> 34 years	0.64 (0.55–0.75) < 0.001	0.57 (0.48–0.68) < 0.001	0.67 (0.56–0.80) < 0.001	0.72 (0.59–0.87) 0.001	0.74 (0.63–0.86) < 0.001	0.85 (0.70–1.04) 0.111	0.77 (0.59–1.00) 0.049	0.85 (0.61–1.18) 0.336
Marital status								
Never married	1.00	1.00	1.00	1.00	1.00	1.00	1.00	1.00
Married/ living with partner	2.72 (1.50–4.90) 0.001	2.98 (1.67–5.29) < 0.001	1.99 (1.09–3.66) 0.026	2.48 (1.37–4.50) 0.003	0.33 (0.24–0.45) < 0.001	0.29 (0.21–0.40) < 0.001	0.31 (0.18–0.53) 0.001	0.30 (0.17–0.51) < 0.001
Previously married	1.09 (0.57–2.01) 0.791	1.22 (0.67–2.24) 0.510	10.6 (0.56–1.99) 0.858	1.32 (0.71–2.45) 0.379	0.20 (0.15–0.27) < 0.001	0.19 (0.14–0.26) < 0.001	0.18 (0.11–0.29) < 0.001	0.18 (0.11–0.30) < 0.001
Education level								
None	1.00	1.00	1.00	1.00	1.00	1.00	1.00	1.00
Primary	5.05 (4.05–6.30) < 0.001	4.57 (0.362–5.77) < 0.001	5.42 (3.90–7.54) < 0.001	4.9 (3.48–6.90) < 0.001	2.03 (1.43–2.89) < 0.001	1.53 (1.04–2.25) 0.029	1.31 (0.72–2.38) 0.373	1.14 (0.58–2.23) 0.708
Secondary & higher	6.65 (5.07–8.73) < 0.001	5.20 (3.86–7.01) < 0.001	5.56 (3.79–8.20) < 0.001	4.14 (2.73–6.28) < 0.001	2.64 (1.84–3.77) < 0.001	1.63 (1.08–2.47) 0.019	2.09 (1.18–3.70) 0.012	1.45 (0.65–3.21) 0.342
Number of children								
2–4	1.00	–	–	–	1.00	1.00	1.00	1.00
> 5	0.71 (0.58–0.77) < 0.001	–	–	–	0.74 (0.62–0.87) < 0.001	0.85 (0.68–1.04) 0.119	1.06 (0.79–1.43) 0.693	1.42 (0.69–2.94) 0.172
Employed in the past year	–	–	–	–	1.20 (1.02–1.42) 0.030	1.27 (1.07–1.51) 0.006	1.15 (0.82–1.62) 0.422	1.32 (0.98–1.78) 0.065
Household wealth index								
Low	1.00	1.00	1.00	1.00	1.00	1.00	1.00	1.00
Middle	1.93 (1.65–2.26) < 0.001	1.52 (1.28–1.80) < 0.001	1.75 (1.41–2.18) < 0.001	1.47 (1.18–1.45) 0.001	1.14 (0.90–1.46) 0.281	1.12 (0.86–1.46) 0.383	1.14 (0.81–1.06) 0.452	1.19 (0.75–1.90) 0.452
High	2.36 (1.97–2.83) < 0.001	1.72 (1.41–2.11) < 0.001	1.88 (1.50–2.36) < 0.001	1.66 (1.27–2.16) < 0.001	1.53 (1.25–1.88) < 0.001	–	1.42 (1.06–1.91) 0.019	1.44 (0.80–2.59) 0.073
Community level factors								
Rural residence	0.81 (0.68–0.98) 0.029	0.78 (0.99–1.48) 0.062	0.83 (0.67–1.03) 0.085	1.11 (0.87–1.41) 0.409	0.83 (0.67–1.01) 0.066	1.14 (0.84–1.55) 0.396	0.82 (0.58–1.31) 0.229	0.99 (0.63–.1.58) 0.985
High poverty level	0.64 (0.51–0.81) < 0.001	–	0.74 (0.27–0.96) 0.023	1.04 (0.74–1.46) 0.821	0.44 (0.40–0.48) < 0.001	0.65 (0.48–0.89) 0.006	1.18 (1.02–1.36) 0.028	1.14 (0.62–2.08) 0.678
High illiteracy level	0.56 (0.46–0.69) < 0.001	0.78 (0.63–0.96) 0.020	0.74 (0.59–0.93) 0.011	0.95 (0.75–1.21) 0.694	0.64 (0.51–0.74) < 0.001	0.81 (0.63–1.02) 0.076	056 (0.40–0.80) 0.001	0.72 (0.45–1.15) 0.173
High unemployment level	–	–	–	–	0.86 (0.63–1.18) 0.353	1.02 (0.74–1.40) 0.914	1.39 (0.86–2.25) 0.181	1.62 (0.94–2.77) 0.080

Married or living with a partner (aOR: 2.48, 95% CI: 1.37–4.50), primary (aOR: 4.9, 95% CI: 3.48–6.90) and secondary or higher level of education (aOR: 4.14, 95% CI: 2.73–6.28) was associated with higher odds for modern contraceptive use among Kenyan women who experienced under-five mortality. Age > 34 years was associated with lower odds (aOR: 0.72, 95% CI: 0.59–0.87). In Zimbabwe, married or living with partner (aOR: 0.30, 95% CI: 0.17–0.51) and previously married (aOR: 0.18, 95% CI: 0.11–0.30) was associated with lower odds for modern contraceptive use (Table 2).

#### Household and community level factors

Middle (aOR: 1.52, 95% CI: 1.28–1.80) and high (aOR: 1.72, 95% CI: 1.41–2.11) household wealth were positively associated with modern contraceptive in Kenyan women who had experienced high-risk births. Similarly, Kenyan women from middle (aOR: 1.47, 95% CI: 1.18–1.54) and high (aOR: 1.66, 95% CI: 1.27–2.16) household wealth who had experienced under-five mortality had higher odds for using modern contraceptives. Community level factors were largely not associated with contraceptive use in both countries for both women who had experienced high-risk births and under-five mortality, with the exception that, in Zimbabwe, high poverty level (aOR: 0.65, 95% CI: 0.48–0.89) was associated with lower odds of contraceptive use among women who had high risk births (Table 2).

#### Multi-level analysis

After adjusting for individual and community level factors, increasing birth order was positively associated with high-risk births in Kenyan women (aOR: 3.14, 95% CI: 2.95–3.34), increasing mother’s age (aOR: 1.02, 95% CI: 1.02–1.04) and middle wealth index quartile (aOR: 1.27, 95% CI: 1.06–1.53). Primary (aOR: 0.77, 95% CI: 0.62–0.96) and secondary education (OR: 0.62, 95% CI: 0.47–0.82) were negatively associated with high-risk births. In Zimbabwe, increasing birth order (aOR: 5.16, 95% CI: 4.69–5.67) and being previously married (aOR: 1.52, 95% CI: 1.22–1.90) were positively associated with high-risk births. Increasing mother’s age (aOR: 0.93, 95% CI: 0.92–0.94), modern contraceptive use (aOR: 0.79, 95% CI: 0.68–0.92), primary (aOR: 0.42, 95% CI: 0.24–0.76) and secondary education (aOR: 0.21, 95% CI: 0.12–0.38) were negatively associated with high-risk births. Community level factors (i.e. place of residence, illiteracy, unemployment and poverty levels) were not significantly associated with high-risk births (Table 3).

**Table 3:** Factors associated with high-risk births at individual and community levels

Characteristic	Kenya				Zimbabwe			
	Empty model	Individual level aOR (95% CI) *p*-value	Community level aOR (95% CI) *p*-value	Individual & community level aOR (95% CI) *p*-value	Empty model	Individual level aOR (95% CI) *p*-value	Community level aOR (95% CI) *p*-value	Individual and community level aOR (95% CI) *p*-value
Fixed effects								
Birth order continuous		3.12 (2.39–3.32) < 0.001		3.14 (2.95–3.34) < 0.001		5.15 (4.66–5.65) < 0.001		5.16 (4.69–5.67) < 0.001
Mothers age Continuous		1.03 (1.02–1.04) < 0.001		1.02 (1.02–1.04) < 0.001		0.93 (0.92–0.94) < 0.001		0.93 (0.92–0.94) < 0.001
Modern contraceptive use		1.12 (0.97–1.30) 0.125		1.09 (0.94–1.26) 0.272		0.80 (0.69–0.93) 0.003		0.79 (0.68–0.92) 0.002
Married/living with partner		1.07 (0.68–1.66) 0.774		1.05 (0.38–1.64) 0.823		1.20 (0.95–1.52) 0.129		1.20 (0.95–1.52) 0.130
Previously married		1.26 (0.79–2.00) 0.332		1.21 (0.76–1.92) 0.426		1.52 (1.22–1.89) < 0.001		1.52 (1.22–1.90) < 0.001
Primary education		0.97 (0.79–1.19) 0.773		0.77 (0.62–0.96) 0.021		0.41 (0.23–0.72) 0.002		0.42 (0.24–0.76) 0.004
Secondary education and above		0.77 (0.60–0.99) 0.047		0.62 (0.47–0.82) 0.001		0.20 (0.11–0.35) < 0.001		0.21 (0.12–0.38) < 0.001
Employed in last 12 months		0.96 (0.81–1.14) 0.670		0.88 (0.74–1.04) 0.130		1.03 (0.90–1.19) 0.650		1.03 (0.89–1.19) 0.718
Middle wealth index		1.27 (1.06–1.53) 0.011		1.27 (1.06–1.53) 0.011		0.97 (0.80–1.15) 0.774		0.98 (0.80–1.20) 0.871
High wealth index		1.05 (0.87–1.25) 0.620		1.02 (0.83–1.26) 0.818		0.98 (0.82–1.16) 0.793		0.98 (0.73–1.15) 0.459
Place of residence (rural)			1.45 (1.33–1.58) < 0.001	1.04 (0.87–1.24) 0.650			2.21 (1.89–2.58) < 0.001	0.86 (0.67–1.14) 0.244
Illiteracy levels (high)			1.49 (1.37–1.62) < 0.001	1.01 (0.85–1.21) 0.895			2.48 (0.35–22.90) 0.327	0.28 (0.02–3.98) 0.347
Unemployment levels (high)			1.11 (0.93–1.31) 0.256	1.09 (0.76–1.58) 0.629			0.77 (0.65–0.91) 0.002	0.93 (0.74–1.17) 0.532
Poverty levels (high)			1.82 (1.63–2.03) < 0.001	0.84 (0.66–1.05) 0.153			1.09 (0.90–6.23) 0.925	0.99 (0.11–9.05) 0.991
Random effects								
Community level Variance	0.39 (0.03)	0.28 (0.08)	0.10 (0.02)	0.21 (0.07)	0.26 (0.04)	0.06 (0.04)	0.06 (0.02)	0.04 (0.04)
ICC	0.11	0.08	0.03	0.06	0.07	0.02	0.02	0.01
PCV	REF	28%	74%	46%	REF	80%	80%	85%
Model fit stats								
Log-likelihood	−13,540	− 3102	−13,127	− 3075	− 5710	− 2649	− 5590	− 2637
AIC	27,083	6228	26,281	6196	11,424	5321	11,210	5323

Under-five mortality in Kenya were positively associated with a higher birth order (aOR: 1.57, 95% CI: 1.15–1.62), only primary school education (aOR: 1.30, 95% CI: 1.07–1.58), and high poverty levels (aOR: 1.33, 95% CI: 1.07–1.64). Higher maternal age aOR: 0.99, 95% CI: 0.98–0.99), using modern contraceptive methods (aOR: 0.79, 95% CI: 0.70–0.89) and living in rural areas (aOR: 0.78, 95% CI: 0.67–0.91) provided protection against under-five mortality (Table 4). In Zimbabwe, an increasing birth order (aOR: 1.81, 95% CI: 1.70–1.92), living with partner/spouse (aOR: 1.42, 95% CI: 1.10–1.84) and being previously married (aOR: 1.30, 95% CI: 1.03–1.1.65) was associated with under-five mortality. Similar to Kenyan women, a protective effect was seen in higher maternal ages (aOR: 0.95, 95% CI: 0.94–0.96), using modern contraception (aOR: 0.71, 95% CI: 0.61–0.83) and living in rural areas (aOR: 0.57, 95% CI: 0.43–0.77). Zimbabwean women with primary (aOR: 0.66, 95% CI: 0.46–0.94), secondary (aOR: 0.65, 95% CI: 0.45–0.93) levels of education and a high wealth index (aOR: 0.76, 95% CI: 0.59–0.98) were less likely to experience under-five child mortality. With the exception of high poverty levels in Kenya (aOR: 0.66, 95% CI: 0.46–0.94) and place of residence in Zimbabwe (aOR: 0.66, 95% CI: 0.46–0.94), community level factors were not significantly associated with under-five mortality (Table 4).

**Table 4:** Factors associated with under-five mortality at individual and community levels

	Kenya				Zimbabwe			
Characteristics	Empty model	Individual level aOR (95% CI) *p*-value	Community level aOR (95% CI) *p*-value	Individual & community level aOR (95% CI) *p*-value	Empty model	Individual level aOR (95% CI) *p*-value	Community level aOR (95% CI) *p*-value	Individual and community level aOR (95% CI) *p*-value
Fixed effects								
Birth order continuous		1.60 (1.54–1.65) < 0.001		1.57 (1.51–1.62) < 0.001		1.79 (1.69–1.90) < 0.001		1.81 (1.70–1.92) < 0.001
Mothers age continuous		0.98 (0.97–0.99) 0.006		0.99 (0.98–0.99) 0.035		0.95 (0.94–0.96) < 0.001		0.95 (0.94–0.96) < 0.001
Modern contraceptive use		0.80 (0.70–0.90) < 0.001		0.79 (0.70–0.89) < 0.001		0.71 (0.61–0.84) < 0.001		0.71 (0.61–0.83) < 0.001
Married/living with partner		1.46 (0.05–2.04) 0.024		1.31 (0.94–1.82) 0.107		1.37 (1.06–1.78) 0.018		1.42 (1.10–1.84) 0.007
Previously married		2.10 (1.48–3.00) < 0.001		1.89 (1.33–2.68) < 0.001		1.16 (0.92–1.47) 0.205		1.30 (1.03–1.65) 0.029
Primary education		1.56 (1.30–1.87) < 0.001		1.30 (1.07–1.58) 0.007		0.62 (0.43–0.86) 0.009		0.66 (0.46–0.94) 0.021
Secondary education and above		1.17 (0.93–1.47) 0.179		0.96 (0.75–1.22) 0.717		0.62 (0.43–0.90) 0.012		0.65 (0.45–0.93) 0.020
Employed in last 12 months		1.30 (1.12–1.50) 0.100		1.25 (1.08–1.45) 0.003		1.13 (0.97–1.33) 0.124		–
Middle wealth index		0.98 (0.83–1.16) 0.841		0.96 (0.82–1.31) 0.649		1.18 (0.95–1.45) 0.131		1.04 (0.83–1.28) 0.736
High wealth index		1.01 (0.86–1.47) 0.179		1.04 (0.87–1.24) 0.670		1.07 (0.87–1.31) 0.521		0.76 (0.59–0.98) 0.034
Place of residence (rural)			1.10 (0.99–1.22) 0.08	0.78 (0.67–0.91) 0.001			1.44 (1.15–1.81) 0.002	0.57 (0.43–0.77) < 0.001
Illiteracy levels (high)			1.28 (1.15–1.43) < 0.001	1.03 (0.88–1.20) 0.716			7.87 (0.93–66.37) 0.058	2.46 (0.24–24.78) 0.445
Unemployment levels (high)			1.05 (0.84–1.31) 0.653	1.01 (0.73–1.39) 0.958			0.86 (.68–1.09) 0.201	1.07 (0.84–1.37) 0.576
Poverty levels (high)			1.74 (1.51–1.99) < 0.001	1.33 (1.07–1.64) 0.009			1.49 (0.20–11.1) 0.695	1.95 (0.30–12.94) 0.484
Random effects								
Community level Variance	0.41 (0.04)	0.25 (0.06)	0.18 (0.03)	0.11 (0.05)	0.28 (0.06)	0.21 (0.06)	0.11 (0.04)	0.06 (0.04)
ICC	0.11	0.07	0.05	0.03	0.08	0.06	0.03	0.02
PCV	REF	39%	56%	73%	REF	25%	61%	76%
Model fit stats								
Log-likelihood	−10,404	− 4064	−10,196	− 3996	− 3091	− 2411	− 3037	− 2359
AIC	20,811	8153	20,419	8038	6186	4845	6104	4768

## Discussion

In this analysis, we described the spatial distribution of contraceptive coverage, and the prevalence of high-risk births and under-five mortality. We also characterised the factors associated with contraceptive use among women who had experienced high-risk births and under-five mortality in Kenya and Zimbabwe. Contraceptive use prevalence, though less than 50% in both countries, was lower in Kenya compared to Zimbabwe. The spatial maps, especially in the Kenyan North-eastern region, show an inverse correlation between prevalence of contraceptives use and under-five mortality as well as high risk births. This confirms findings from other published studies [5–7]. However, other regions for example Nyanza, which reported the highest contraceptive rate (41%), also reported high rates of high-risk births (26%) and under-five mortality (20%) which could be explained by other factors such as high HIV prevalence in this region compared to the rest of the country [23]. In Zimbabwe, the pattern was less clear, for instance Mashonaland central province had the highest prevalence of contraceptive use as well as higher rates of high-risk births and under five mortality. This unexpected finding may be explained by the fact that the province is predominantly rural with limited healthcare services [24]. It is also plausible that the higher contraceptive prevalence is a result of increased efforts by the ministry of health to reduce the unmet need for contraceptive methods in order to address the high-risk births and under five mortality [25, 26].

At an individual level, in both countries, higher levels of education were significantly associated with higher contraceptive usage in women who had experienced high-risk births and under-five mortality. This finding suggests that more educated women were most likely to understand the benefits of family planning using contraceptives as confirmed in a report from a study in Kenya [14]. Kenyan women, who were married or living together with a partner were significantly more likely to use contraception. However, this was the opposite for Zimbabwe, where married women or women living with partners were less likely to use modern contraception. The reason for this difference is unclear, but could be attributed to cultural variations between the two countries. For example, in Zimbabwe reports suggest that using certain contraceptive methods (such as condoms) in marriage could be regarded as evidence that the woman has more than one partner [27].

At a household level, among Kenyan women who had experienced high-risk births in both countries, women from middle or higher household wealth status were at least one and half times more likely to be using contraceptive compared to women from low household incomes. This could be explained by the fact that women who have financial resources have better access to sexual reproductive health services including contraceptives [28, 29]. It is also supported by our finding that, at community level in Zimbabwe, higher levels of poverty were associated with reduced odds of contraceptive usage.

Multi-level analysis showed that in Zimbabwe, contraceptive use was protective against both high risk births and under-five mortality whilst in Kenya it was only significantly protective against under-five mortality. The effect of contraception use is two-fold as the proportion of high-risk births are reduced and as a result, a decline in child mortality is seen [30]. We also found that maternal education was associated with reduced odds of high risk births and under-five mortality in both countries even though this was more evident in Zimbabwe. It is hypothesised that education protects against high-risk births because young women spend more years in school delaying delivery before 18 years, become more aware of available methods of contraception and tend to be employed, thus have income which can improve their access to health care including contraceptive services [28, 29, 31]. Maternal education provides autonomy in decision making which increases the mother’s ability to access health care for children in a household, thus ultimately contributing to the reducing under-five mortality [32]. We also found that high-risk births were positively associated with increasing maternal age and birth-order. This is keeping in line with literature that shows this association [10, 11]. As confirmed in other studies, a higher maternal age increased child survival in both countries. Younger mothers may lack social and psychological support needed to meet the requirements for childcare [32, 33].

Community level factors, with the exception of high poverty levels in Zimbabwe were not significantly associated with contraceptive use among women who had experienced high-risk births or under-five mortality. This finding was in contradiction with a report from Chama-Chiliba and colleagues which highlighted the strong association between community level factors and uptake of antenatal services [15]. The reason for this difference is unclear but could be due the heterogeneity of the group variable for example, the urban areas are generally characterised by better access to sanitation, nutrition and healthcare, but the proliferation of slums in most African cities often mean that a significant proportion of the urban dwellers face overcrowded living conditions, inadequate sanitation and limited access to quality healthcare including access to contraception services.

The main limitation of this study is that it is cross sectional, therefore associations observed do not imply causality. Secondly, the DHS are conducted independently within countries and thus not measured at the same time which limits the contemporaneous cross-national comparisons to some extent. Thirdly, most of the health measures in DHS are based on self-report or proxy report and thus may suffer from recall and social desirability bias. Fourthly, the analysis of factors associated with contraceptive use was limited to women who had experienced high risk births or under-five mortality and this may have introduced bias in the modelling process by excluding other groups such as women with low and medium risk births. Lastly, contraceptive use was current, whilst under-five mortality and high risk births referred to the past 5 years. It may be that if both measures were provided on the same time scales, findings may have differed. Despite these limitations, DHS has several strengths such as: greater population coverage, standardized data collection procedures across countries which are consistent over time and therefore allowing comparability across different settings. Additionally, the multistage probabilistic sampling design of DHS allowed for the multi-level analyses that we conducted to explore community and individual level factors that influenced the outcomes of interest. Furthermore, our analysis provides results from two SSA countries with different settings, thus the findings could be extrapolated to other countries within the region.

### Public health and policy implications

The low contraceptive prevalence rates in both countries suggests that more effort is required to improve uptake of contraception and alleviate unmet needs. The increased contraception coverage would in turn contribute to the reduction of under-five mortality and possibly high-risk births. Improving education and poverty levels of girls and women in the SSA region might help improve contraceptive prevalence rates, decrease high-risk births and under-five mortality in the region.

## Conclusions

There is need to increase contraceptive coverage due to the positive benefits associated with it such as reduction of unplanned pregnancies and high risk-births as well as under-five mortality. Increasing access to education and subsequent economic opportunities for women in sub-Saharan Africa could help achieve multiple improved health benefits.

## Additional files

Additional file 1: **Figure S1.** Distribution of regional contraceptive coverage, prevalence of high-risk births and under-five mortality in Kenya and Zimbabwe. Show the prevalence of contraceptive use, high-risk births and under-five mortality in Kenya and Zimbabwe stratified by regional/provincial level. (PNG 175 kb)

Additional file 2: **Figure S2.** Proportion of women using contraception in urban vs. rural regions on Kenya and Zimbabwe. Compares urban and rural contraceptive use in Kenya and Zimbabwe stratified by regional/provincial level. (PNG 176 kb)

Additional file 3: **Figure S3.** Proportion of high-risk births in urban vs. rural regions in Kenya and Zimbabwe. Compares urban and rural high-risk births prevalence in Kenya and Zimbabwe stratified by regional/provincial level. (PNG 189 kb)

Additional file 4: **Figure S4.** Proportion of under-five child mortality in urban vs. rural regions of Kenya and Zimbabwe**.** Compares urban and rural under-five child mortality prevalence in Kenya and Zimbabwe stratified by regional/provincial level. (PNG 180 kb)

## Abbreviations

AIC: Akaike Information Criterion
AOR: Adjusted odds ratio
CI: Confidence Intervals
DHS: Demographic Health Survey
ICC: Intra Community Correlation Coefficient
IQR: Interquartile range
MMR: Maternal mortality ratio
PCV: Proportional Change in Community Variance
PSU: Primary Sampling Unit
SDG: Sustainable Development Goal
SSA: Sub-Saharan Africa
U-5MR: Under-5 mortality rate
