# Supplementary figures and images for: Use of contraceptives, high risk births and under-five mortality in Sub Saharan Africa: evidence from Kenyan (2014) and Zimbabwean (2011) demographic health surveys
Source: BMC Womens Health. 2018 Oct 24;18:173. doi: 10.1186/s12905-018-0666-1 (PMC6201505; doi:10.1186/s12905-018-0666-1)

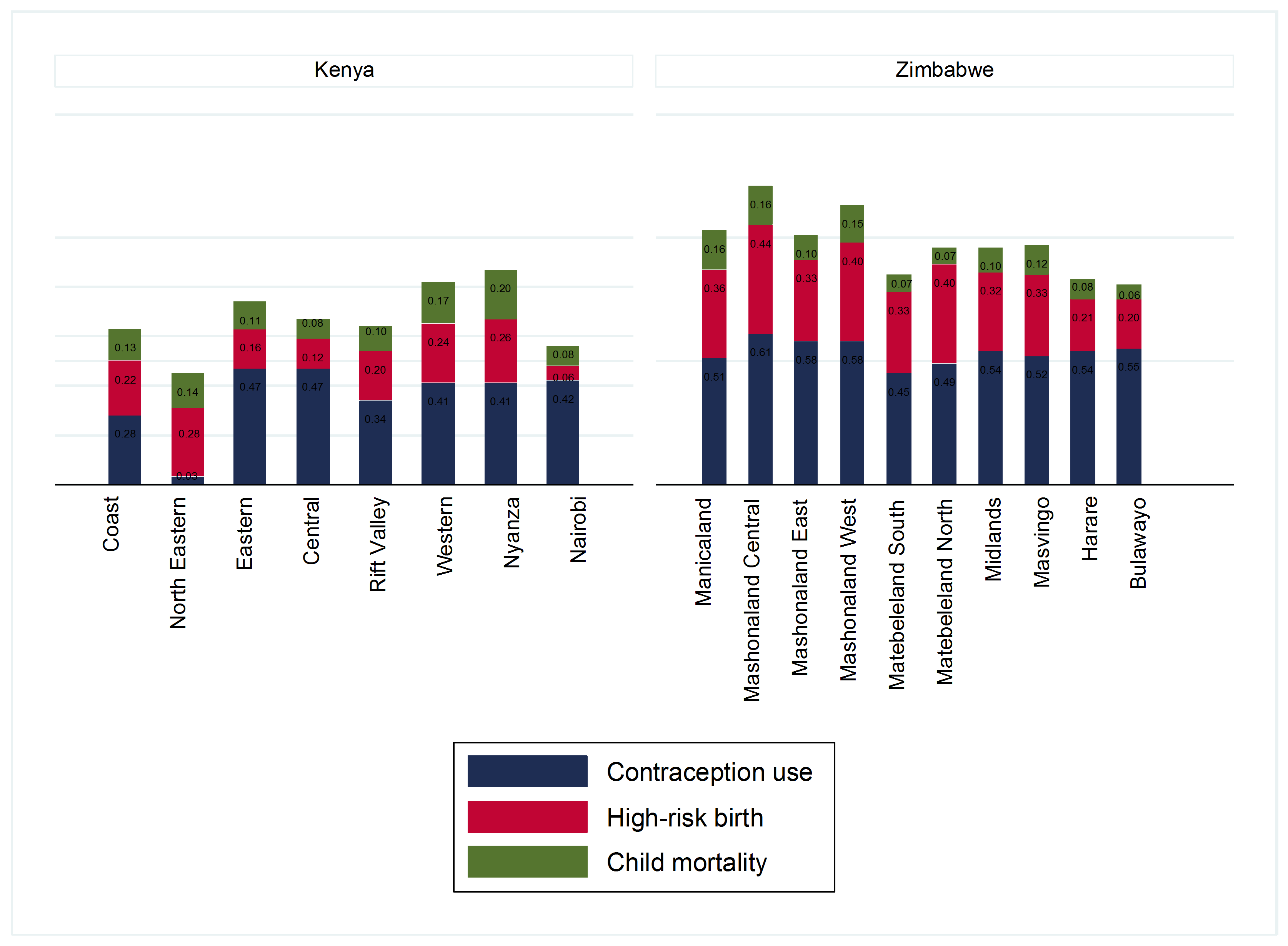

Supplement: Supplementary file 1 — Figure S1. Distribution of regional contraceptive coverage, prevalence of high-risk births and under-five mortality in Kenya and Zimbabwe. Show the prevalence of contraceptive use, high-risk births and under-five mortality in Kenya and Zimbabwe stratified by regional/provincial level. (PNG 175 kb) [file 12905_2018_666_MOESM1_ESM.png]

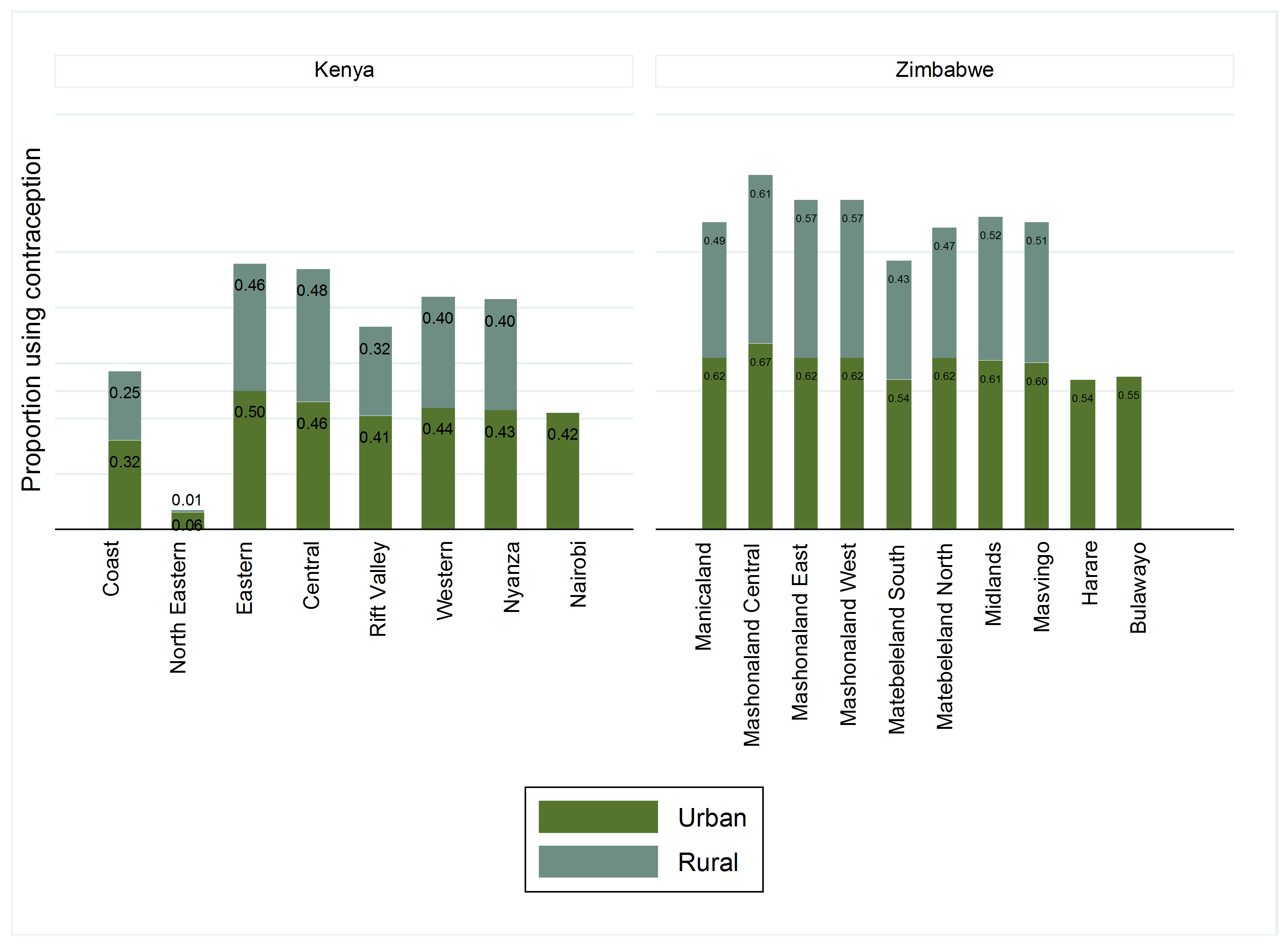

Supplement: Supplementary file 2 — Figure S2. Proportion of women using contraception in urban vs. rural regions on Kenya and Zimbabwe. Compares urban and rural contraceptive use in Kenya and Zimbabwe stratified by regional/provincial level. (PNG 176 kb) [file 12905_2018_666_MOESM2_ESM.png]

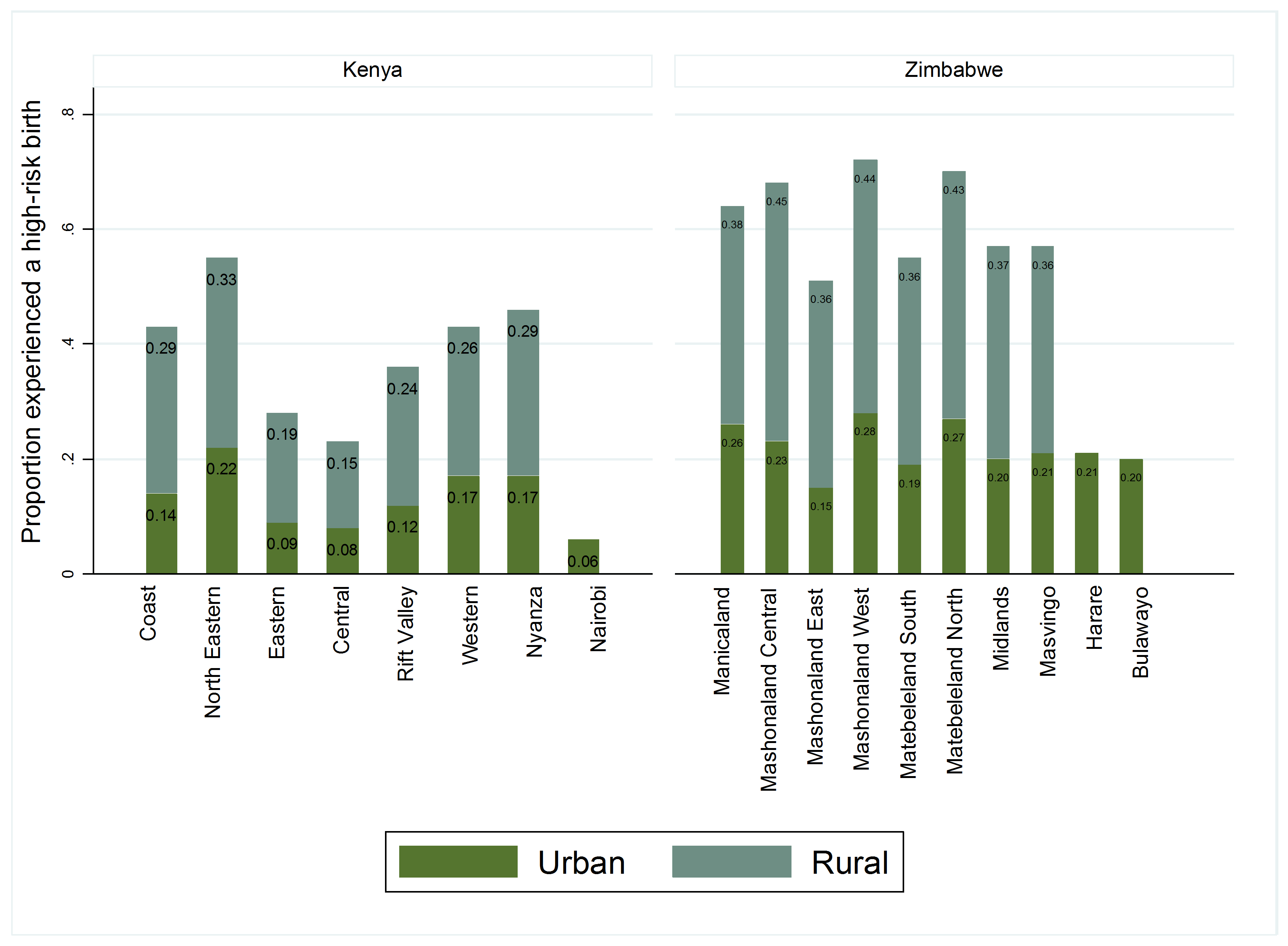

Supplement: Supplementary file 3 — Figure S3. Proportion of high-risk births in urban vs. rural regions in Kenya and Zimbabwe. Compares urban and rural high-risk births prevalence in Kenya and Zimbabwe stratified by regional/provincial level. (PNG 189 kb) [file 12905_2018_666_MOESM3_ESM.png]

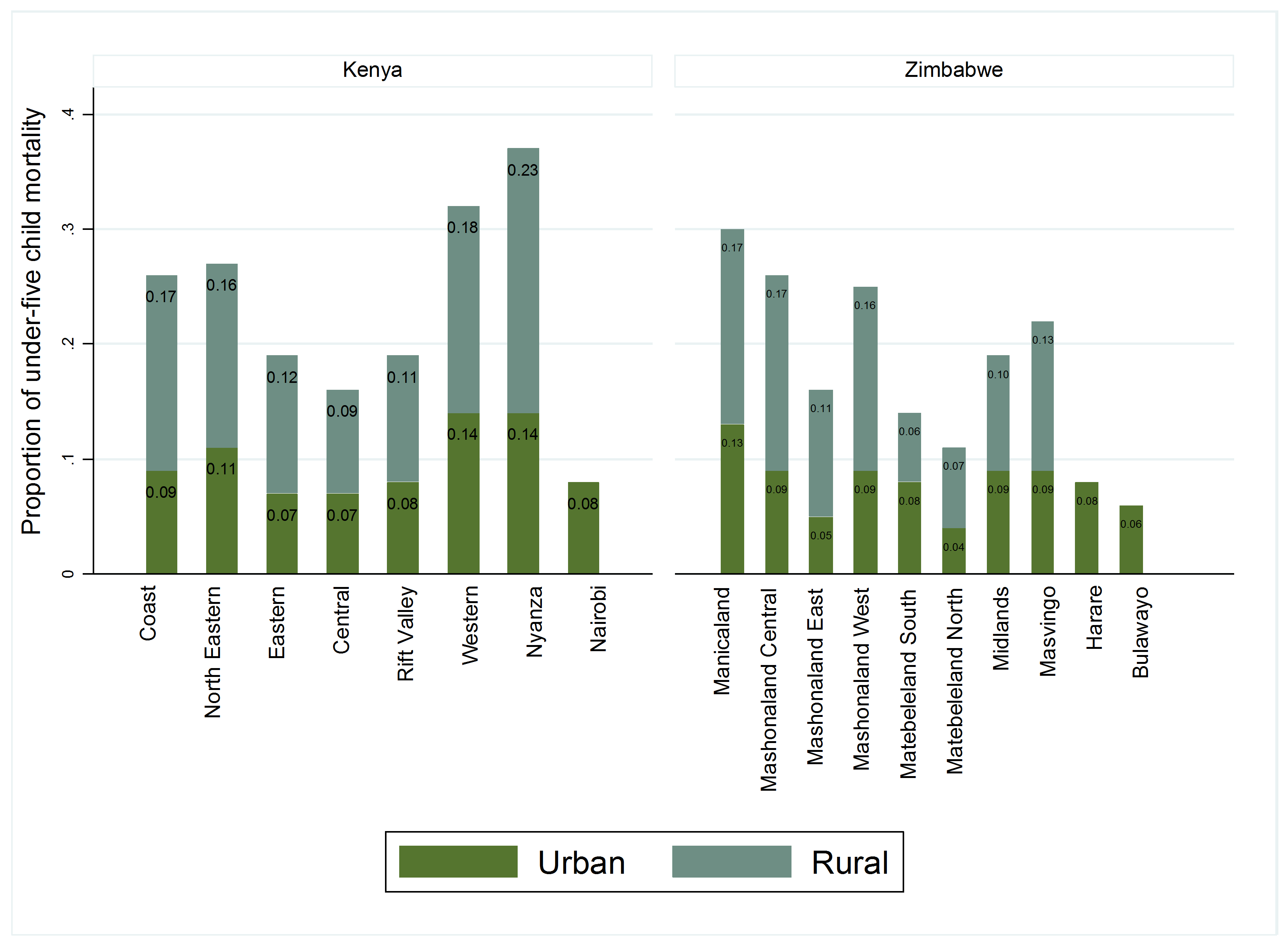

Supplement: Supplementary file 4 — Figure S4. Proportion of under-five child mortality in urban vs. rural regions of Kenya and Zimbabwe. Compares urban and rural under-five child mortality prevalence in Kenya and Zimbabwe stratified by regional/provincial level. (PNG 180 kb) [file 12905_2018_666_MOESM4_ESM.png]
